# Multigenerational Heat Selection Enhancing Thermal Acclimation and Transcriptional Response of Hsps to Heat Stress in *Spodoptera frugiperda* Male Adults

**DOI:** 10.3390/insects16080860

**Published:** 2025-08-18

**Authors:** Zhi-Xiao Zhang, Qing-Yi Zhao, Yu Song, Guo-Yun Yu, Wen Fu, Jin Xu

**Affiliations:** 1 Yunnan Provincial Key Laboratory for Conservation and Utilization of In-Forest Resource, Southwest Forestry University, Kunming 650224, China; zhangzhixiao@yafg.ac.cn (Z.-X.Z.); zqy0133@126.com (Q.-Y.Z.); sy3167632311@163.com (Y.S.); yuguoyun1063431541@162.com (G.-Y.Y.); 2 Yunnan Academy of Forestry and Grassland, Kunming 650201, China; 3 Laboratory of Forest Disaster Warning and Control in Yunnan Province, College of Forestry, Southwest Forestry University, Kunming 650224, China; 4 Plant Conservation and Quarantine Station of Yunnan Province, Kunming 650034, China; fuwenyn@yeah.net

**Keywords:** *Spodoptera frugiperda*, heat stress response, heat acclimation, heat shock proteins, metabolism, transcription

## Abstract

In this study, using a multigenerational heat selection design, we investigated the possible pattern of thermal acclimation and the background mechanism in *Spodoptera frugiperda*. Results show that heat selection significantly shortened the larval and pupal developmental duration, which was maintained in the four tested generations. Significant costs to reproduction were found in the first two generations, but they recovered in the following generations. Heat-selected adults exhibited significantly higher survival rates under extremely high temperatures, and the survival rate increased with subsequent generations. Transcriptomic analysis between heat-selected and non-selected male adults from the first generation showed that heat stress did not induce the upregulation of any heat shock protein (Hsp) genes; in the fourth generation, however, heat stress resulted in the upregulation of a great number of Hsps, indicating a positive correlation between Hsp expression and heat tolerance. Further analysis also found differential expression in other genes associated with heat resistance, such as cuticle-, antioxidant-, and detoxification-related genes. Moreover, quite a number of stress response GO terms were enriched, such as response to heat and other abiotic stimuli. GO enrichment also showed that DEGs were enriched to many more terms related to metabolism. KEGG enrichment revealed that relatively more disease-, metabolism-, and immunity-associated pathways were enriched. The number of disease pathways increased with the selection temperature in the first generation, whereas it decreased with subsequent generations. This study offers new information for the understanding of the thermal acclimation process and mechanism of moth species, and facilitates the prediction and management of this major pest.

## Simple Summary

The study of heat stress and acclimation can provide a theoretical basis for predicting and controlling insect pests. Here, we show that multigenerational heat selection significantly shortened the developmental duration of *Spodopera frugiperda*. Heat selection caused significant costs to reproduction in only the first two generations. Heat selection significantly enhanced the heat tolerance of adults. Moreover, multigenerational heat selection significantly enhanced the heat sensitivity and regulation potential of Hsps in male adults. These results suggest that this pest has strong heat tolerance potential, and the influence of global warming on its population development and spread may be limited.

## 1. Introduction

Over the past few decades, global temperature changes have intensified, and extremely high temperatures have frequently occurred [1]. Ambient temperature is a vital factor limiting the adaptive distribution of insects, determining the distribution and fundamental niche of insect species [2,3,4]. Insects, as small ectothermic animals, are highly sensitive to temperature changes [5]. Research on insect thermal stress and reaction not only expands our understanding of species-adaptive evolution but also provides a theoretical basis for the prediction and prevention of insect pests [2,6].

Invasive insect species are a significant ecological issue, as they have a destructive impact on biodiversity, agriculture, and ecosystems [7]. Invasive insects often exhibit remarkable adaptability to temperature fluctuations, enabling their establishment in new environments [8]. Additionally, climate warming may expand their suitable habitats, increasing invasion risk [9,10]. Understanding these thermal adaptations is crucial for predicting and managing invasive insect outbreaks under global climate change. Lepidoptera is an economically significant insect order due to its important role in ecological conservation and agriculture. The diversity of adaptive responses to different thermal niches in Lepidoptera is equally significant [9]. Lepidopterans have evolved various physiological and behavioral adaptations to survive high temperatures. However, the mechanisms related to heat adaptation and tolerance and their relationship with survival and reproductive fitness in Lepidoptera still need further clarification [9]. Heat stress can negatively affect the development, survival, and reproduction of insects, which may be a consequence of cellular stress caused by high temperatures, ultimately leading to death [4,11]. A large number of studies have found that mild temperature hardening and long- or short-term exposure of insects to sublethal high temperatures enhances their heat tolerance and leads to reversible and irreversible physiological changes [11,12]. Heat adaptation refers to the process by which organisms gradually enhance their tolerance to high temperatures through repeated exposure to such environments, which is an adaptive mechanism for insects to survive under heat stress [11,12,13]. For example, pre-acclimation of the adult codling moth *Cydia pomonella* under 37 °C significantly improved its survival under extreme temperature from 20% to 90% under 43 °C [14]. In the common cutworm, *Spodoptera litura*, pre-exposure at mild temperature stress significantly increased heat tolerance but decreased reproductive fitness in adults [15]. In the fall armyworm, *Spodoptera frugiperda*, adult thermotolerance is associated with age, sex, and mating status [16]. Pre-acclimation by 20~30 °C in *S. frugiperda* revealed that the thermotolerance of first instars and adults increased with the increase in acclimation temperature [17].

It is worth noting that studies in different insect species have revealed that their survival rates improved under lethal temperatures after a few generations of heat exposure, indicating transgenerational heat acclimation [11,18,19,20]. For example, in the moth *Cnaphalocrocis medinalis*, multigenerational heat selection increased heat tolerance of larvae [18]. However, a study in the Angoumois grain moth, *Sitotroga cerealella*, found that both heat and fasting acclimation treatments showed negative effects on the offspring’s thermotolerance, with continuous dynamic heat (28~38 °C) acclimation having stronger effects than acute static (38 °C) acclimation [12]. These results further imply the plasticity and critical mechanisms of heat adaptation and tolerance of insects from a transgenerational perspective.

The mechanism by which organisms adapt to heat stress remains enigmatic [11] and may involve epigenetic memory, DNA methylation, and gene transcription and translation [11,18,21]. DNA methylation plays a key role in regulating gene expression, genomic stability, and cellular differentiation [22]. In response to thermal stress, insects undergo a complex process involving changes in gene expression and methylation [11,23]. Thermal stress in insects typically leads to upregulation of heat shock proteins (Hsps), Hsp-related genes, and Hsp transcription factors [24]. When insects are subjected to external stress, their cellular homeostasis may be disrupted, and the functions of proteins and other molecules may also be affected. Hsps are a highly conserved family of molecular chaperones that play critical roles in cellular stress response and protein homeostasis by assisting in the correct folding of newly synthesized polypeptides and repairing misfolded proteins damaged by heat or other stressors [25]. Generally, Hsps are categorized into four families: Hsp90, Hsp70, Hsp60, and small Hsps (sHsps), based on their molecular weight [26]. Within the Hsp families, Hsp90 and Hsp70 are the most universally described Hsps in the thermal stress response and tolerance of insects [24]. In *Aphis gossypii*, heat stress induced marked upregulation of three *Hsp70* genes [27]. The expression of *Hsp70* and *Hsp90* increased dramatically after heat shock in the macropterous *Nilaparvata lugens* [28,29] and the fruit fly *Bactrocera dorsalis* [30]. In *S. litura*, mild thermal stress induced significant upregulation of *Hsp90*, *Hsc90*, *Hsp70*, and *Hsc70* [15]. A previous study in *S. frugiperda* by RNA-seq found that short-term heat stress induced large-scale upregulation of different Hsp families in both male and female adults [31]. A recent study in *S. frugiperda* also found that heat stress induced prominent upregulation of *Hsp70* in larval brain [32].

In addition to heat shock proteins, other processes and genes may also contribute to the heat tolerance of insects. Desiccation is one of the major detrimental effects of thermal stress in insects because of their small size and associated large ratio of surface/volume [33]. The cuticle is considered the major structure by which terrestrial insects prevent water loss [34,35,36]. Factors shaping cuticular permeability include the composition and amount of epicuticle lipids, which are the primary waterproofing components [34,37]; cuticular proteins (CPs) [38]; and the structure and composition of the cuticle under the lipid layer [34]. Recent studies also revealed that aquaporins may play a role in insect thermal tolerance [39]. Aquaporins are a class of transmembrane proteins that may participate in the adaptive response of insects to high- and low-temperature stress by regulating the flow of water and glycerol [39,40]. Moreover, heat stress may also induce the upregulation of genes encoding detoxification and antioxidant enzymes [41], and thermal stimuli detection channels such as TRPA1 and TRPV1 [42,43]. For example, heat stress significantly increased the production of primary antioxidative enzymes in *Mythimna separate* (Lepidoptera: Noctuidae) such as catalase (CAT), glutathione-S-transferase (GST), and superoxide dismutase (SOD) [44]. In *S. frugiperda*, heat stress induced the upregulation of two TRPA1 genes in female adults and the downregulation of one TRPA1 gene in male adults [31]. These results have indicated physiological and molecular foundations of heat acclimation in insects.

The fall armyworm, *Spodoptera frugiperda* (Lepidoptera: Noctuidae), is an extremely harmful, long-distance migratory agricultural pest [45]. This moth pest, originally from the Americas, was first discovered in the southwest region of China at the end of 2018 and has since rapidly spread across vast areas of China [46]. This moth pest can cause significant economic losses to corn production. It is estimated that the potential annual economic losses caused by this pest in China range from approximately USD 17.3 billion to 52.1 billion [47]. Moreover, its remarkable foliar damage, high fecundity, and strong pesticide resistance have triggered excessive use of chemical pesticides, even some banned and hazardous chemicals [48,49,50]. Environmentally friendly management methods are required for better control of this highly harmful insect pest [45]. Based on the above achievements on heat stress and adaption mechanisms in insects, we hypothesize that thermal acclimation is a gradual process not only for heat tolerance but also for gene-level response and regulation. To examine this hypothesis, we investigated the process of thermal acclimation by fitness tests and transcriptomic analysis using a multigenerational heat selection design in *S. frugiperda*. We also discussed the costs and trade-offs of heat acclimation and possible mechanisms.

## 2. Materials and Methods

### 2.1. Insects

*S. frugiperda* larvae were collected in July 2022 on corn plants in a corn field near Dongchuan town in Yunnan Province, China. Larvae were then reared under 27 ± 1 °C, 60–80% RH (relative humidity), and a 14:10 h L:D (light:dark) photoperiod on an artificial diet [51]. When preparing the artificial diet, 100 g of soybean meal, 100 g of wheat bran, 30 g of casein, 40 g of yeast powder, and 30 g of agar powder were added to 1 L of distilled water and immediately stirred evenly with a spatula. Then, the mixture was placed in a steamer and steamed for 2 h. After cooling the mixture to 50 °C, 3 g of potassium sorbate and 3 g of VC were added and stirred thoroughly with a spatula to obtain the final diet. The final diet was stored at 4 °C for future use. Mature pupae were sexed based on abdominal characteristics, and then male and female pupae were caged separately [52]. Adult eclosion was recorded daily, and eclosed male and female moths were caged separately to ensure age and virginity. Adults were reared with 10% honey solution.

### 2.2. Multigenerational Heat Selection

Multigenerational heat exposure was conducted throughout the entire lifecycle of *S. frugiperda* (including egg, larval, pupal, and adult stages) by exposing the insects to different temperatures for different durations (namely, 32 °C for 2 h, 32 °C for 4 h, 37 °C for 2 h, and 37 °C for 4 h, forming four treatments) once each day for four successive generations (F1~F4). Heat treatments were performed during scotophase. The insects were maintained at 27 °C as above for the remaining time. The heat selection temperatures were set according to previous studies [12,18] and local temperature (Dongchuan: July, 18~32 °C, average 24 °C). Normally reared insects under 27 °C (without heat exposure) were used as a control. Insects were fed as above for all treatments. The treatments were conducted in an artificial climate incubator (BIC-400; Shanghai Boxun, Shanghai, China) that can control temperature, photoperiod (14:10 h L:D), and RH (60–80%). The temperature and RH were also verified by using handheld temperature and humidity meters. In addition, to avoid desiccation-induced stress, 10% honey solution was provided during heat treatments and the whole adult stage.

The developmental periods and adult reproductive fitness of each generation were measured. The developmental periods of larvae and male and female pupae were measured by using 60 randomly selected insects for each treatment (n = 60). Adult males and females of 3 d-old insects (reproductively mature) were collected randomly from each treatment and were paired for mating in plastic boxes (15 cm wide, 25 cm long, and 8 cm high), with one pair per box. Each box was placed a folded (zigzag fashion) paper strip (15 × 20 cm) for oviposition, and a 10% honey solution was provided as food. The laid eggs were collected and placed in a culture dish (8.5 × 1.5 cm) for incubation under the above conditions. The number of hatched eggs was counted after 4 days of incubation. Twenty pairs of adult insects were set for each treatment for their lifetime to determine egg production and hatching rate (n = 20).

### 2.3. Survival Test of the Heat-Acclimated Adults Exposed to Heat Stress

The 3 d-old female and male adults from each generation of the above treatments were randomly collected and subjected to heat stress at 42 °C or 45 °C for 2 h. Their deaths were recorded 12 h after heat stress under 27 °C. Three replicates were used for each treatment, with 20 males or females being used for each replicate.

### 2.4. Effect of Heat Acclimation on the Transcription of Adult Males

#### 2.4.1. Treating and Sampling

Three d-old male moths from the F1 and F4 generations of the above 32 °C 4 h (T1) and 37 °C 4 h (T2) selection lines were randomly collected and stressed at 42 °C for 2 h (HS). Insects were sampled immediately after stress. Six insects were pooled to obtain a biological replicate, and three biological replicates were set per treatment. Collected samples were frozen immediately in liquid nitrogen and then were stored at −80 °C before use.

#### 2.4.2. Sequencing and Assembly

Total RNA was isolated from the above samples using the TRIzol reagent (Invitrogen Inc., Calsbad, CA, USA) according to the manufacturer’s instructions. RNA concentration was measured using a spectrophotometer (Implen Inc., Westlake Village, CA, USA), and RNA purity was assayed by using a Qubit RNA Assay Kit (Life Technologies Inc., Frederick, MD, USA). Then, RNA integrity was determined using the RNA Nano 6000 Assay Kit (Agilent Inc., Palo Alto, CA, USA). Sequencing libraries were constructed using 10 μg of RNA from each sample by using the NEBnext Ultra RNA Library Prep Kit for Illumina (New England BioLabs Inc., San Diego, CA, USA). The constructed libraries were paired-end sequenced on the Illumina HiSeq 4000 platform (Illumina Inc., San Diego, CA, USA), which produces 125 bp/150 bp paired-end reads.

High-quality clean read datasets were achieved from the raw reads through the following procedure by using fastp software (v0.23.4). Firstly, the adapter was trimmed from the reads, and reads containing > 5% undeterminable bases were removed. Secondly, low-quality reads (containing > 20% suspect bases and base quality < 10) were deleted. Lastly, both ends of the reads were evaluated, and unreliable ends containing > 3 suspect bases were trimmed. The Q20, Q30, and GC contents of the obtained clean read datasets were calculated as well. The clean datasets were mapped to the reference genome sequence of *S. frugiperda* (assembly AGI-APGP CSIRO Sfru_2.0) [53] by using the Hisat2 software (v2.2.1).

#### 2.4.3. Differential Expression Analysis and Functional Enrichment

Gene expression levels were determined by using TPM (transcripts per million), and DESeq2 (v1.56.1) was used to analyze the differential expression between treatments. The *p*-value (significance threshold) from multiple tests was corrected by the *q*-value, and then *q* < 0.05 with |log2(foldchange)| > 1 was used as the threshold to determine the significant differences of gene expression [54]. The 95% CI of DEG numbers of different comparison groups was also calculated by using the binom.confint procedure in the R language (v4.5.1).

The GOSeq software (v2.12) was used to conduct GO enrichment analysis, and the KOBAS program (v2.1.1) was used to implement KEGG enrichment analysis of DEGs (differentially expressed genes), with *q* < 0.05 being used as the threshold to determine significantly enriched GO terms and KEGG pathways.

#### 2.4.4. Validation of RNA-Seq

RNA-seq results were verified using qPCR. RNAs were extracted from samples as above, and the cDNAs were prepared using a PrimeScript RT reagent kit (Takara, Beijing, China). *Actin* (LOC118279073) was used as the reference gene. PCR was conducted using gene-specific primers (Table S1) and the QuantStudio 7 Flex System (Thermo Fisher Scientific Inc., Carlsbad, CA, USA) in a volume of 25 μL with the following program: 95 °C for 30 s, then 40 cycles of 95 °C for 5 s and 60 °C for 30 s, followed by dissociation. Three biological replicates were set for each sample, and each biological replicate was run in three technical replicates. Standard curves of target and reference genes were constructed by 5× serial dilution of cDNA to determine PCR efficiencies. PCR signal specificity was determined by melting curve analysis, and the 2^−ΔΔCT^ method [55] was run to calculate the relative quantities of the target gene.

### 2.5. Statistics

The data on developmental period, reproduction, survival rate, and qPCR were analyzed by one-way ANOVA, followed by Fisher’s LSD test for pairwise comparisons. Percentage data were arcsin square root-transformed. Before ANOVA, data were tested for homogeneity and normality of variances across the treatments using Levene’s test and the Shapiro–Wilk test, respectively. The effect size *η*^2^ and its 95% CI of ANOVA were also reported. All analyses were performed using SPSS 16.0. The rejection level was set at *α* < 0.05, and all values were reported as mean ± SE.

## 3. Results

### 3.1. Effect of Heat Selection on Development and Reproduction

**Figure 1:**
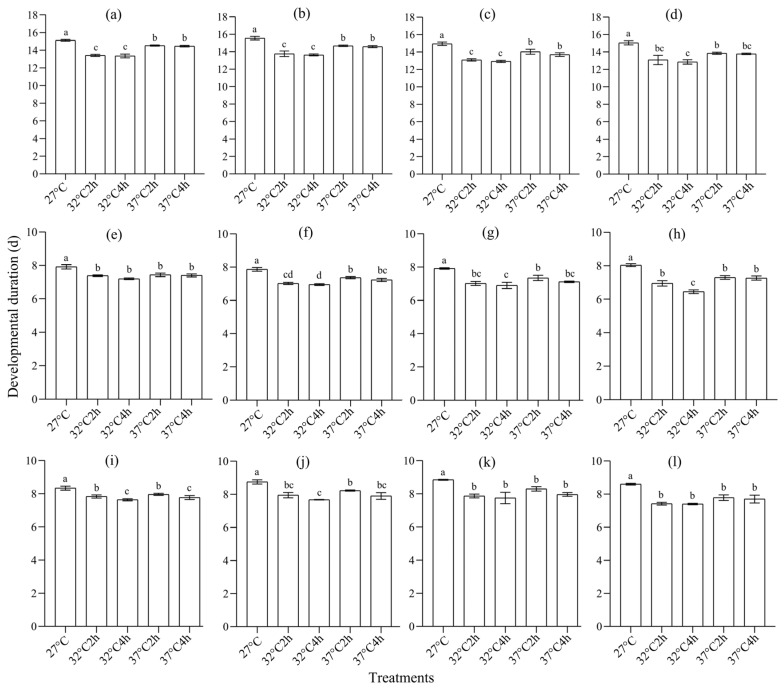
The effect of heat selection on the developmental duration of *S. frugiperda*. (**a**–**d**) The developmental duration of larvae of generations F1–F4, respectively; (**e**–**h**) the developmental duration of female pupae of generations F1–F4, respectively; (**i**–**l**) the developmental duration of male pupae of generations F1–F4, respectively. In each subgraph, bars with different letters are significantly different (*p* < 0.05).

Heat selection significantly shortened the developmental period of larvae [F1: *F*_4,295_ = 24.624, *p* < 0.0001, *η*^2^ = 0.250 (95% CI: 0.175, 0.308); F2: *F*_4,295_ = 23.911, *p* < 0.0001, *η*^2^ = 0.245 (0.170, 0.302); F3: *F*_4,295_ = 20.421, *p* < 0.0001, *η*^2^ = 0.217 (0.143, 0.274); F4: *F*_4,11_ = 26.938, *p* < 0.0001, *η*^2^ = 0.268 (0.191, 0.325); Figure 1a–d)], female pupae [F1: *F*_4,295_ = 7.938, *p* < 0.0001, *η*^2^ = 0.097 (0.042, 0.143); F2: *F*_4,295_ = 14.401, *p* < 0.0001, *η*^2^ = 0.163 (0.096, 0.217); F3: *F*_4,295_ = 17.631, *p* < 0.0001, *η*^2^ = 0.193 (0.122, 0.249); F4: *F*_4,11_ = 39.890, *p* < 0.0001, *η*^2^ = 0.351 (0.274, 0.408); Figure 1e–h)], and male pupae [F1: *F*_4,295_ = 5.48, *p* < 0.0001, *η*^2^ = 0.069 (0.022, 0.11); F2: *F*_4,295_ = 16.29, *p* < 0.0001, *η*^2^ = 0.181 (0.111, 0.236); F3: *F*_4,295_ = 19.73, *p* < 0.0001, *η*^2^ = 0.211 (0.138, 0.268); F4: *F*_4,11_ = 23.99, *p* < 0.0001, *η*^2^ = 0.245 (0.170, 0.303); Figure 1i–l)]. A post hoc LSD test showed that, in most cases, larvae and pupae under 32 °C for 2 h and those under 32 °C for 4 h usually had the shortest period (*p* < 0.05), followed by those under 37 °C for 2 h and those under 37 °C for 4 h, while those under 27 °C (controls) had the longest period (*p* < 0.05) (Figure 1).

**Figure 2:**
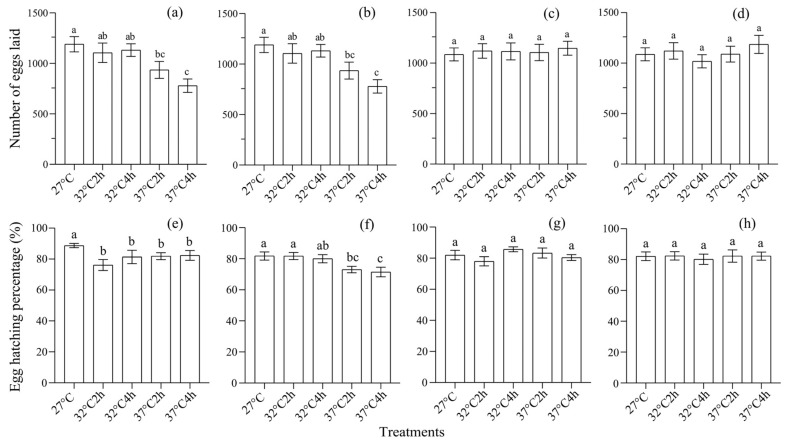
The effect of heat selection on the reproductive fitness of *S. frugiperda*. (**a**–**d**) The number of eggs laid by the adults of generations F1–F4, respectively; (**e**–**h**) the egg hatching rate of the adults from generations F1–F4, respectively. In each subgraph, bars with different letters are significantly different (*p* < 0.05).

Heat selection also significantly affected the fecundity (F1: *F*_4,95_ = 4.644, *p* < 0.001, *η*^2^ = 0.164 (0.041, 0.247); F2: *F*_4,95_ = 6.086, *p* < 0.0001, *η*^2^ = 0.204 (0.071, 0.291); Figure 2a,b) and egg hatching rate (F1: *F*_4,95_ = 3.040, *p* < 0.05, *η*^2^ = 0.089 (0, 0.157); F2: *F*_4,95_ = 3.714, *p* < 0.01, *η*^2^ = 0.139 (0.025, 0.219); Figure 2e,f) of adults from the first two generations, and showed a temparture- and exposure duration-dependent effect. A post hoc LSD test showed that control adults usually had the higest reproduction, followed by those under 32 °C for 2 h and those under 32 °C for 4 h, while those under 37 °C for 2 h and those under 37 °C for 4 h had the lowest reproduction (*p* < 0.05) (Figure 2a,b,e,f). However, no significant difference was found in the third and fourth generations regarding fecundity (F3: *F*_4,95_ = 0.18, *p* > 0.05, *η*^2^ = 0.008 (0, 0.012); F4: *F*_4,95_ = 0.05, *p* > 0.05, *η*^2^ = 0.002 (0, 0); Figure 2c,d) or egg hatching rate (F3: *F*_4,95_ = 1.37, *p* > 0.05, *η*^2^ = 0.05 (0, 0.1); F4: *F*_4,95_ = 0.19, *p* > 0.05, *η*^2^ = 0.005 (0, 0); Figure 2g,h).

### 3.2. Survival of Heat-Acclimated Adults Under Extreme Temperature

**Figure 3:**
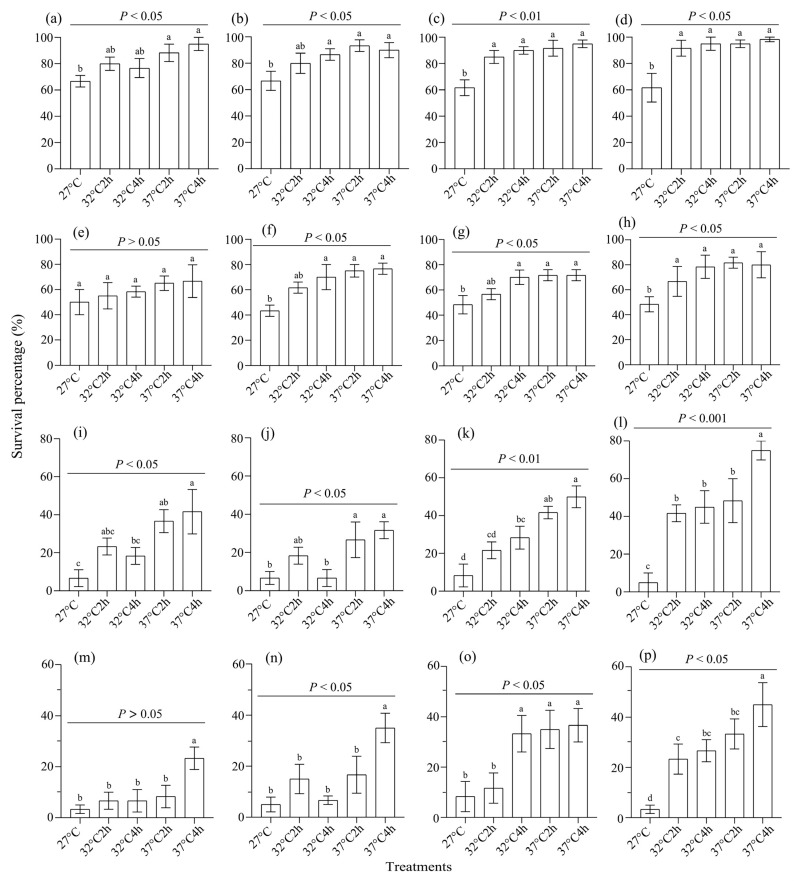
The effect of heat selection on the survival rate of *S. frugiperda* adults under extreme temperatures. (**a**–**d**) The survival rate of female adults from generations F1–F4 under 42 °C for 2 h, respectively; (**e**–**h**) the survival rate of male adults from generations F1–F4 under 42 °C for 2 h, respectively; (**i**–**l**) the survival rate of female adults from generations F1–F4 under 45 °C for 2 h, respectively; (**m**–**p**) the survival rate of male adults from generations F1–F4 under 45 °C for 2 h, respectively. In each subgraph, bars with different letters are significantly different (*p* < 0.05).

Heat-acclimated female and male adults exhibited significantly higher survival rate under extreme heat stress of either 42 °C for 2 h (female: F1, *F*_4,11_ = 3.588, *p* < 0.05, *η*^2^ = 0.612 (0.036, 0.692); F2, *F*_4,11_ = 3.030, *p* < 0.05, *η*^2^ = 0.495 (0, 0.596); F3, *F*_4,11_ = 7.829, *p* < 0.01, *η*^2^ = 0.646 (0.081, 0.719); F4, *F*_4,11_ = 5.942, *p* < 0.05, *η*^2^ = 0.636 (0.067, 0.711); male: F1, *F*_4,11_ = 0.56, *p* > 0.05, *η*^2^ = 0.188 (0, 0.285); F2, *F*_4,11_ = 5.053, *p* < 0.05, *η*^2^ = 0.624 (0.051, 0.701); F3, *F*_4,11_ = 3.913, *p* < 0.05, *η*^2^ = 0.601 (0.022, 0.683); F4, *F*_4,11_ = 3.475, *p* < 0.05, *η*^2^ = 0.419 (0, 0.53); Figure 3a–h) or 45 °C for 2 h (female: F1, *F*_4,11_ = 4.319, *p* < 0.05, *η*^2^ = 0.665 (0.108, 0.734); F2, *F*_4,11_ = 3.9, *p* < 0.05, *η*^2^ = 0.609 (0.032, 0.69); F3, *F*_4,11_ = 9.898, *p* < 0.01, *η*^2^ = 0.758 (0.264, 0.807); F4, *F*_4,11_ = 11.149, *p* < 0.001, *η*^2^ = 0.831 (0.437, 0.865); male: F1, *F*_4,11_ = 1.95, *p* > 0.05, *η*^2^ = 0.438 (0, 0.547); F2, *F*_4,11_ = 5.457, *p* < 0.05, *η*^2^ = 0.647 (0.083, 0.720); F3, *F*_4,11_ = 4.177, *p* < 0.05, *η*^2^ = 0.599 (0.019, 0.681); F4, *F*_4,11_ = 6.902, *p* < 0.01, *η*^2^ = 0.793 (0.342, 0.835); Figure 3i–p). A post hoc LSD test showed that control adults usually had the lowest survival rates, followed by those under 32 °C for 2 h and those under 32 °C for 4 h, while those under 37 °C for 2 h and those under 37 °C for 4 h had the highest survival rates (*p* < 0.05) (Figure 3).

### 3.3. Effect of Heat Selection and Heat Stress on the Transcription of Male Adults

#### 3.3.1. RNA Sequencing and Assembly

RNA-seq achieved 42,280,000~50,690,000 clean reads for each of the 18 libraries. The Q20, Q30, and mapped ratios were 95.20–95.60%, 92.50–93.02%, and 69.94–76.41%, respectively (Table S2). Pearson’s coefficient indicated lower correlations among treatments, whereas higher correlations among replicates (Table S3) and PCA (principal component analysis) indicated that biological replicates grouped together (Figure S1), confirming the reproducibility of RNA-seq and replicates.

#### 3.3.2. Overview of Transcriptional Changes

In comparison with the controls, T1F1-HS induced 175 (66 up, 109 down) DEGs, T1F4-HS induced 558 (313 up, 245 down) DEGs, T2F1-HS induced 335 (180 up, 155 down) DEGs, and T2F4-HS induced 385 (199 up, 186 down) DEGs (Figure S2; Table S4).

In the T1 groups, different generations shared 19 common DEGs, and in the T2 groups, different generations shared 18 common DEGs (Figure S3a,b). In the F1 groups, different treatments shared 39 common DEGs, and in the F4 groups, different generations shared 113 common DEGs (Figure S3c,d).

**Figure 4:**
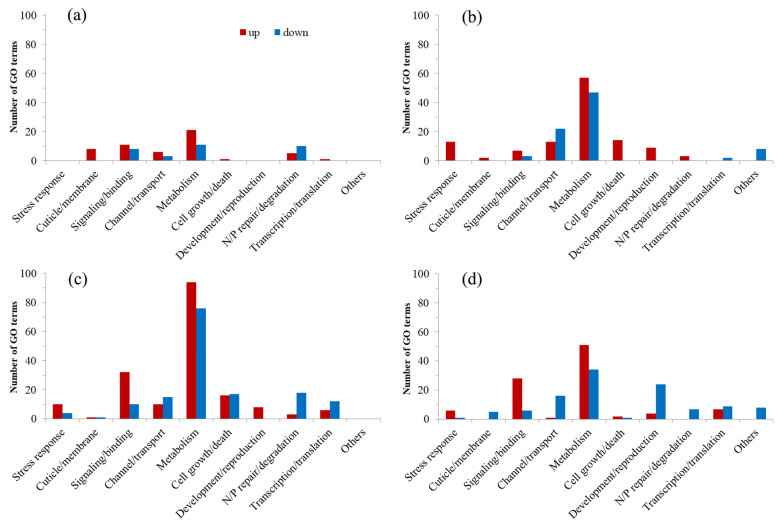
Enriched GO terms. (**a**–**d**) Enriched terms of T1F1-HS vs. CKF1-HS, T2F1-HS vs. CKF1-HS, T1F4-HS vs. CKF4-HS, and T2F4-HS vs. CKF4-HS, respectively. Red columns refer to terms enriched in upregulated DEGs, while blue columns refer to terms enriched in downregulated DEGs. N/P repair/degradation refers to nucleotide/protein repair/degradation.

**Figure 5:**
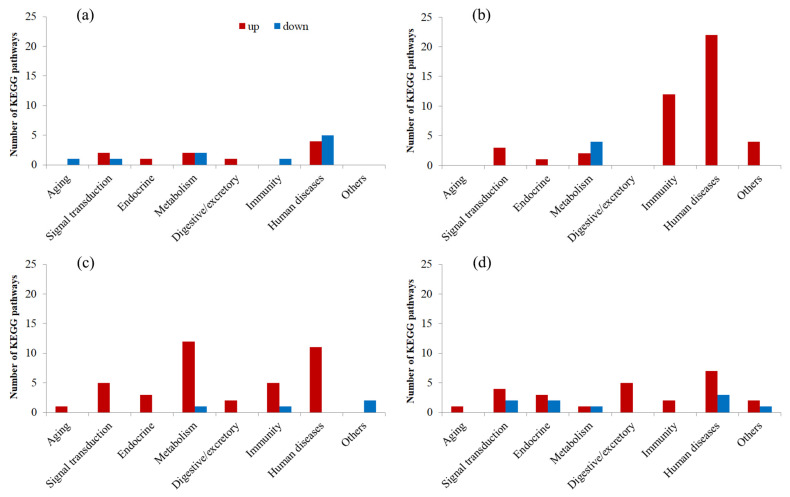
Enriched KEGG pathways. (**a**–**d**) Enriched pathways of T1F1-HS vs. CKF1-HS, T2F1-HS vs. CKF1-HS, T1F4-HS vs. CKF4-HS, and T2F4-HS vs. CKF4-HS, respectively. Red columns refer to pathways enriched in upregulated DEGs, while blue columns refer to pathways enriched in downregulated DEGs.

The abovementioned upregulated and downregulated DEGs from different comparison groups were submitted for GO and KEGG enrichment analyses, respectively. Because significantly (*q* < 0.05) enriched terms/pathways were few for all comparison groups and even nonexistent for some groups, all significantly (*q* < 0.05) and marginally significantly (*p* < 0.05) enriched terms and pathways were used for the following analyses (Tables S5 and S6). For a better understanding and summary, all these terms and pathways were clustered into 10 and 8 groups based on their functions, respectively (Figure 4 and Figure 5). Based on the above analysis, we delved into and elaborated on the important molecular changes related to heat stress as follows:

#### 3.3.3. Transcriptional Changes in the First Generation

T1F1-HS vs. CKF1-HS upregulated DEGs were enriched to 53 terms and 10 pathways, including 8 cuticle/membrane-, 11 signaling/binding-, 6 channel/transport-, and 21 metabolism-related terms; 1 cell growth/death-related term; 5 nucleotide/protein (N/P) repair/degradation-related terms; and 1 transcription/translation-related term, as well as 2 signal transduction-related pathways, 1 endocrine-related pathway, 2 metabolism-related pathways, 1 digestive/excretory-related pathway, and 4 human disease-related pathways. Downregulated DEGs were enriched to 32 terms and 10 pathways, including 8 signaling/binding-, 3 channel/transport-, 11 metabolism-, and 10 N/P repair/degradation-related terms, as well as 1 aging- and 1 signal transduction-related pathway, 2 metabolism-related pathways, 1 immunity-related pathway, and 5 human disease-related pathways (Figure 4a and Figure 5a; Tables S5 and S6).

T2F1-HS vs. CKF1-HS upregulated DEGs were enriched to 118 terms and 44 pathways, including 13 stress response-, 2 cuticle/membrane-, 7 signaling/binding-, 13 channel/transport-, 57 metabolism-, 14 cell growth/death-, 9 development/reproduction-, and 3 N/P repair/degradation-related terms, as well as 3 signal transduction-related pathways, 1 endocrine-related pathway, 2 metabolism-related pathways, and 12 immunity- and 22 human disease-related pathways, plus 4 others (including a Vasopressin-regulated water reabsorption pathway). Downregulated DEGs were enriched to 82 terms and 4 pathways, including 3 signaling/binding-, 22 channel/transport-, 47 metabolism-, and 2 transcription/translation-related terms, plus 8 others, as well as 4 metabolism-related pathways (Figure 4b and Figure 5b; Tables S5 and S6).

**Figure 6:**
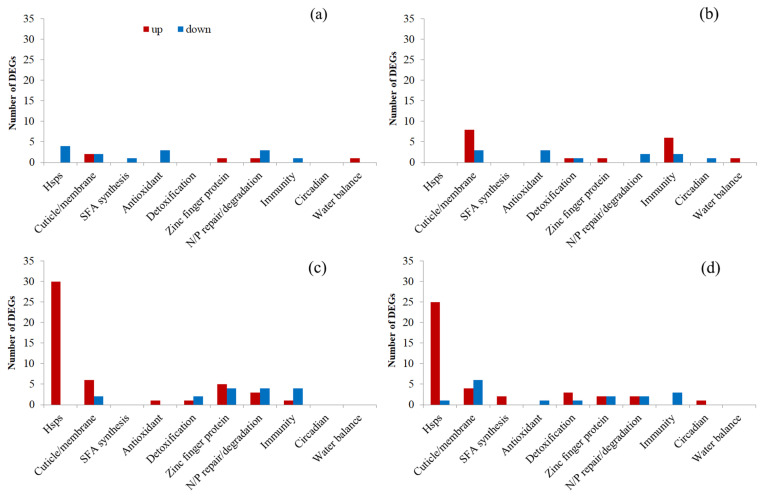
Specific DEGs that may have been directly associated with heat stress resistance. (**a**–**d**) DEGs of T1F1-HS vs. CKF1-HS, T2F1-HS vs. CKF1-HS, T1F4-HS vs. CKF4-HS, and T2F4-HS vs. CKF4-HS, respectively. Red columns refer to upregulated DEGs, while blue columns refer to downregulated DEGs. N/P repair/degradation refers to nucleotide/protein repair/degradation; SFA synthesis refers to saturated fatty acid synthesis.

**Figure 7:**
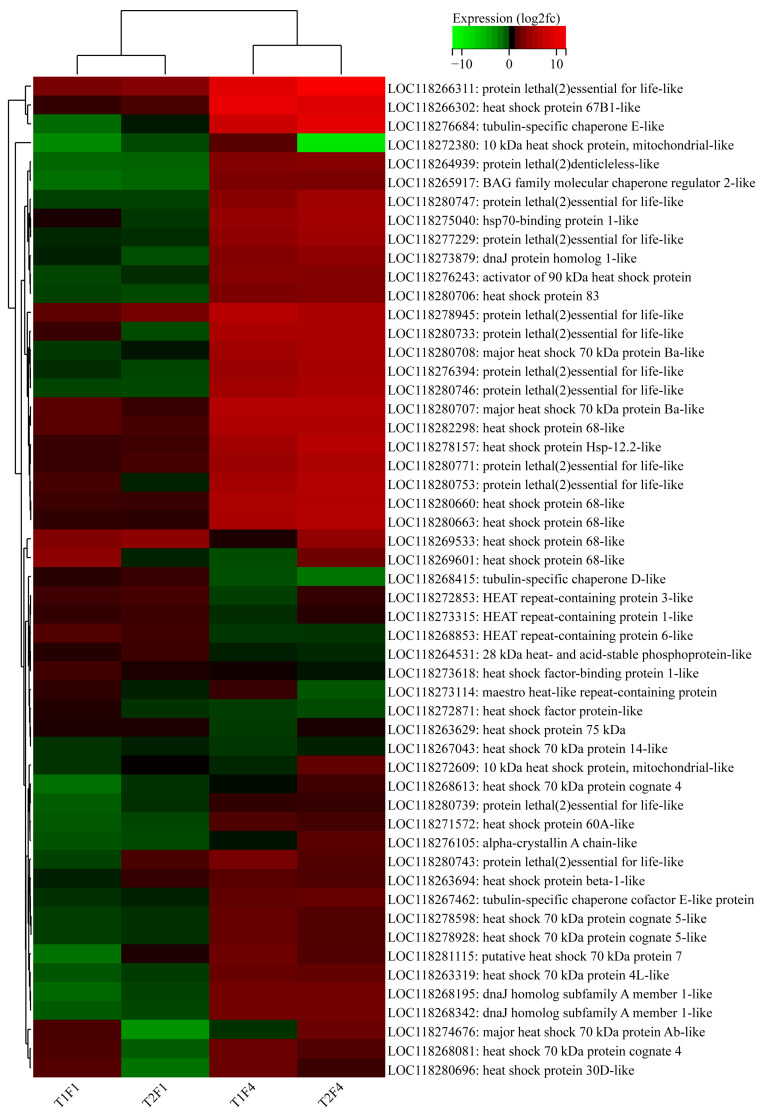
Heatmap of Hsp-related genes from the four comparison groups. T1F1, T2F1, T1F4 and T2F4 refer to T1F1-HS vs. CKF1-HS, T2F1-HS vs. CKF1-HS, T1F4-HS vs. CKF4-HS, and T2F4-HS vs. CKF4-HS, respectively.

Moreover, definite DEGs that might have been directly associated with heat stress reaction and resistance were also identified and illustrated. Few or no Hsp-related DEGs were found in F1 heat-acclimated male adults, with 4 downregulated DEGs in the T1F1-HS vs. CKF1-HS group and no DEGs in the T2F1-HS vs. CKF1-HS group (Figure 6a,b and Figure 7; Table S7). There were more or less 0–11 other resistance-related DEGs found in heat-acclimated male adults (Figure 6a,b and Figure S4; Table S7).

#### 3.3.4. Transcriptional Changes in the Fourth Generation

T1F4-HS vs. CKF4-HS upregulated DEGs were enriched to 180 terms and 39 pathways, including 10 stress response-related terms, 1 cuticle/membrane-related term, and 32 signaling/binding-, 10 channel/transport-, 94 metabolism-, 16 cell growth/death-, 8 development/reproduction-, 3 N/P repair/degradation-, and 6 transcription/translation-related terms, as well as 1 aging-related pathway and 5 signal transduction-, 3 endocrine-, 12 metabolism-, 2 digestive/excretory-, 5 immunity-, and 11 human disease-related pathways. Downregulated DEGs were enriched to 153 terms and 4 pathways, including 4 stress response-related terms, 1 cuticle/membrane-related term, and 10 signaling/binding-, 15 channel/transport-, 76 metabolism-, 17 cell growth/death-, 18 N/P repair/degradation-, and 12 transcription/translation-related terms, as well as 1 metabolism and 1 immunity-related pathway, plus 2 others (Figure 4c and Figure 5c; Tables S5 and S6).

T2F4-HS vs. CKF4-HS upregulated DEGs were enriched to 99 terms and 25 pathways, including 6 stress response- and 28 signaling/binding-related terms, 1 channel/transport-related term, and 51 metabolism-, 2 cell growth/death-, 4 development/reproduction-, and 7 transcription/translation-related terms, as well as 1 aging-related pathway, 4 signal transduction- and 3 endocrine-related pathways, 1 metabolism-related pathway, and 5 digestive/excretory-, 2 immunity-, and 7 human disease-related pathways, plus 2 others. Downregulated DEGs were enriched to 111 terms and 9 pathways, including 1 stress response-related term; 5 cuticle/membrane-, 6 signaling/binding-, 16 channel/transport-, and 34 metabolism-related terms; 1 cell growth/death-related term; and 24 development/reproduction-, 7 N/P repair/degradation-, 9 transcription/translation-related terms, plus 8 others, as well as 2 signal transduction- and 2 endocrine-related pathways, 1 metabolism-related pathway, and 3 human disease-related pathways, plus 1 other (Figure 4d and Figure 5d; Tables S5 and S6).

Specifically, many more Hsp-related DEGs were found in F4 heat-acclimated male adults, with 30 upregulated DEGs and no downregulated DEGs in the T1F4-HS vs. CKF4-HS group, and 25 upregulated DEGs and only 1 downregulated DEG in the T2F4-HS vs. CKF4-HS group (Figure 6c,d and Figure 7; Table S7). Also, there were more or less 0–10 other resistance-related DEGs found in heat-acclimated male adults (Figure 6c,d and Figure S4; Table S7).

From the overall perspective of the first and fourth generations, the expression of Hsp-related genes found in *S. frugiperda* was indistinctive (log2fc close to 0) in F1 of both T1 and T2, with only 4% (4/106) showing significant changes, whereas most (>70%) of these genes showed remarkable upregulation in F4, with 57% (30/53) of T1 and 47% of T2 being significantly upregulated (*q* < 0.05) (Figure 7). In other resistance-related genes, relatively few of them were significantly changed in F1, with 16% (14/87; 5 up, 6 down) for T1 and 31% (27/87; 17 up, 10 down) for T2; relatively more of them were significantly changed in F4, with 38% (33/87; 17 up, 16 down) for T1 and 33% (29/87; 14 up, 15 down) for T2 (Figure S4). Generation-specific expression changes were observed in these genes, where quite a number of genes, such as some immunity-related genes (e.g., LOC118270354: attacin-A-like, LOC118279278: gloverin-like), showed obvious upregulation in F1, while many other genes, such as some detoxification-related genes (e.g., LOC118271639: glutathione S-transferase 2-like, LOC118270316: cytochrome P450 4V2-like), showed remarkable upregulation in F4 (Figure S4). A few genes, such as LOC118264651 (E3 ubiquitin-protein ligase SIAH1-like) and LOC118271398 (CAPA peptides-like), showed upregulation in generations F1 and F4 (Figure S4).

### 3.4. Validation of RNA-Seq Data

**Figure 8:**
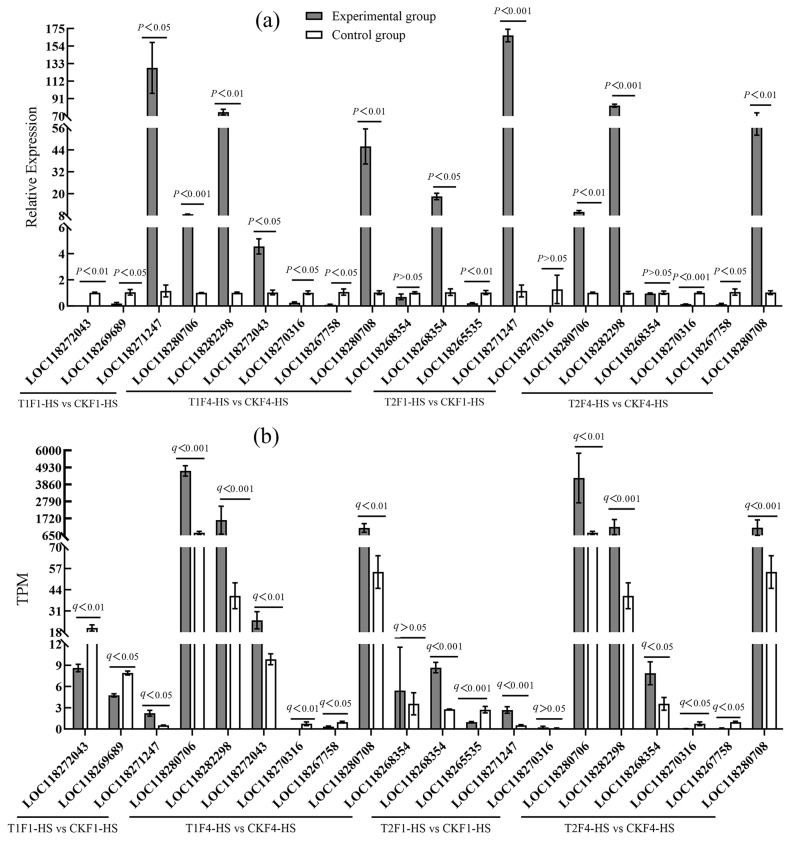
Validation of RNA-seq sequencing data by qPCR in *S. frugiperda.* (**a**) Relative expression levels of target genes measured by qPCR, and (**b**) their expression levels measured by RNAseq. Error bars are standard error (SE).

A total of 20 genes (including DEGs and non-DEGs of Hsps, P450, and other heat tolerance-related genes; Table S1) were selected from the four comparison groups for validation of the accuracy of RNA-seq. The results show that the expression levels of almost all (except LOC118268354 in T2F4-HS vs. CKF4-HS) target genes from qPCR were similar to the results from the RNA-seq analysis (Figure 8), suggesting that the RNAseq results were dependable.

## 4. Discussion

Climate change affects all living organisms on Earth. Insects are typical ectothermic animals with strong adaptability to the environment and can have more survival opportunities in changing environments [6,56,57]. High temperature usually shortens the developmental period of insects. The developmental periods of various stages of *Galleria mellonella* were gradually shortened with the increase in temperature in the range of 27–35 °C [58]. After heat exposure at 35 °C, the development rate of *Anagrus nilaparvatae*, a rice planthopper egg parasitoid wasp, was significantly greater than that of the control population [59]. Here, we found that the developmental periods of *S. frugiperda* larvae and pupae were significantly shortened after high-temperature selection, and this phenomenon was maintained in the four tested generations. Elevated temperatures enhance insect growth primarily by boosting metabolic rates, increasing enzyme activity (e.g., digestive and respiratory enzymes), and accelerating energy production [24]. However, this rapid growth often trades off with reduced lifespan due to oxidative stress. Moreover, high developmental temperature generally produces small individuals due to enhanced metabolism and fast development, which may consequently reduce the reproductive fitness of adults [10].

In the present study, the effect of heat selection on reproduction showed a different pattern, where significant costs to fecundity and egg hatching were indeed found in the first two generations, whereas these parameters recovered in the following two generations. Similarly, it was found in *C. medinalis* that short-term thermal acclimation led to a decrease in reproductive ability, but after two to three generations of thermal acclimation, the reproductive ability significantly improved [18]. Heat shock-incurred reproductive costs may be due to multiple pathways, such as germ cell damage, hormonal disruption, and trade-offs between survival and reproduction [4,11,15]. The trade-off between survival and reproduction is a fundamental concept in life history theory [60]. Organisms allocate limited resources to either maintaining their own survival or investing in reproduction [61,62]. Increased survival efforts may reduce reproductive capacity due to depleting reserves, and vice versa [62,63,64], while the recovery of reproduction after a few generations of thermal selection may be due to physiological and molecular adaptation [11,13,18].

It is widely recognized that thermal acclimation is able to modify insects’ thermotolerance [11,12,18]. For instance, the survival of adult *Sitophilus zeamais* adults under mortal high temperatures could be enhanced through prior heat exposure [65]. In *C. medinalis*, the heat-acclimated larvae (through multigenerational heat selection) showed significantly greater survival than the control larvae under lethal high temperatures [18]. Multigenerational heat selection also increased the thermal acclimation of the multivoltine midge *Chironomus riparius* [19] and the invasive Mediterranean *Bemisia tabaci* [20]. More prominently, the present study demonstrated that heat-acclimated *S. frugiperda* female and male adults exhibited significantly greater survival rate under extreme heat stress of either 42 °C or 45 °C for 2 h, and the survival rate increased with subsequent generations. The fast adaption of *S. frugiperda* to high-temperature stress suggests that this pest has strong thermal tolerance potential, and the impact of global warming on its population development may be limited. The enhanced heat tolerance in *S. frugiperda* and other insect pests may expand pest populations, prolong damage periods, increase the number of generations, and disrupt the natural predator–prey balance, raising agricultural costs [9].

Field studies in many insect species have shown that environmental temperature stress usually constitutes a certain degree of selection pressure in organisms, which leads to the rapid evolution of biological adaptability under specific conditions [66,67,68]. Insects with strong migratory and dispersal abilities are more susceptible to environmental temperature stress, resulting in strong selection pressure and evolutionary dynamics [66,68]. Laboratory studies on temperature stress and responses in insects can provide a theoretical basis for predicting their adaptive development and dispersal [6,69]. For example, in the damselfly *Ischnura elegans*, a lab-based study showed that prior exposure to low temperature, simulating a cold snap, improved future cold tolerance [70], which is consistent with field studies that have shown that larvae at the expansion front evolved an improved cold tolerance [71]. However, thermal response and acclimation of insects can be complicated in the wild due to interactions with multiple environmental stressors, such as humidity, pathogens, and predators [69,72,73]. Therefore, further field studies under multiple factor conditions are required to test the adaptation and expansion of *S. frugiperda*.

Many invasive species possess broad thermal tolerance ranges or rapid acclimation abilities, allowing them to survive in diverse climates [8], and climate warming may further expand their suitable habitats and invasion risks [9]. However, there are numerous factors that inhibit the successful colonization of a new habitat [7]. Remarkably, the genetic composition of the founding population (including the number of additive genetic variations) and its ability to quickly adapt to new environments are important factors for the successful colonization of new habitats and the expansion of the distribution range after establishment [7,9,10].

In response to thermal stress, insects undergo a complex process involving changes in gene expression and methylation [11,23,32]. Recent studies based on high-throughput sequencing have also deepened our understanding of heat stress and acclimation mechanisms. In *C. medinalis*, after five generations’ acclimation at 39 °C, heat-acclimated versus control larvae after exposure to 41 °C exhibited 1160 DEGs, including upregulated *Hsp27*, *Hsp70*, and *CRYAB* genes [74]. In the predatory mite, *Neoseiulus barkeri*, comparative transcriptome and proteome analyses between a high temperature-adapted strain and a conventional strain obtained 5374 DEGs and 500 differentially expressed proteins [75]. Among them, some processes, such as higher level of Hsps, were conserved, whereas many protective enzymes, such as SOD, GST, P450, and peroxidase, displayed downregulation [75].

In *S. frugiperda*, a previous study has shown that a short duration of heat stress (40 °C for 3 h, compare to 27 °C for 3 h) induced large-scale transcriptional changes in adults (2186 and 1276 DEGs in females and males, respectively), including the upregulation of many (16 in females and 26 in males) Hsp-related genes in both sexes [31]. In the present study, on the basis of multigenerational heat selection, comparative transcriptomic analysis between heat-selected and non-selected male adults from the first generation showed that heat stress did not induce the upregulation of any Hsps. However, in males from the fourth generation, heat stress resulted in the upregulation of many Hsp-related genes in heat-selected male adults, with 30 and 25 upregulated Hsp DEGs in the T1F4-HS vs. CKF4-HS group and the T2F4-HS vs. CKF4-HS group, respectively. Moreover, the heatmap also exhibited that the expression of all Hsp-related genes found in *S. frugiperda* was unremarkable in F1 male adults, while most (>70%) of them showed remarkable upregulation in F4 males. These results are consistent with the adaption test that the heat tolerance of adults increased with the increase in heat selected generations, affirming the positive correlation of Hsp expression and heat tolerance in *S. frugiperda* male adults. Similarly, in *C. medinalis*, the expression of *Hsp70* and *Hsp90* in the heat-acclimated larvae was significantly greater than in the control larvae under extreme heat stress at 37 °C and 41 °C [18]. These results suggest that multigenerational heat selection increased the heat sensitivity and regulation potential of Hsps and further confirm that Hsps play vital roles in insect heat stress response and tolerance.

A recent study in *C. medinalis* further found that heat acclimation correlated with the upregulation of oxidoreductase- and SOD-encoding genes [11]. Moreover, larvae of the heat-acclimated strain exhibited higher DNA methylation levels, while pupae from recovery generations displayed lower methylation, indicated by the downregulation of two methyltransferase-encoding genes and the upregulation of a demethylase gene at high temperatures [11]. In the present study, however, no methyltransferase- or demethylase-related DEGs were found in heat-acclimated males. Therefore, further studies using whole-genome bisulfite sequencing (this is a shortcoming in the current study) are required to test heat stress-induced methylation in *S. frugiperda*.

In this study, further analysis also revealed differential expression in other genes that may be directly associated with heat stress response and resistance, such as cuticle-, antioxidant-, detoxification-, and DNA repair-related genes. However, these genes’ transcriptional changes were not consistent, showing both upregulation and downregulation in each gene category. Further overall analysis of these genes by heatmap revealed that relatively few of them were significantly changed in F1, whereas more of them were significantly changed in F4. Generation-specific expression changes were found in these genes, such as some immunity-related genes having obvious upregulation in F1 males, while some detoxification-related genes exhibited remarkable upregulation in F4 males. The differential expression of these genes may be associated with the process of physiological adaptation to heat stress in *S. frugiperda* and other insects [18,19,74]. In addition, some other genes showed upregulation in both F1 and F4 males, such as the E3 ubiquitin–protein ligase SIAH1 and CAPA peptides. E3 ubiquitin–protein ligase SIAH1 is a crucial regulator of protein stability and cellular signaling pathways [76]. CAPA peptides are anti-diuretic factors in insects that regulate ion–water homeostasis via Malpighian tubules [77]. Maintaining water balance in insects in hot environments is particularly important, as insects have a large ratio of surface area to volume [78].

In *S. litura*, heat acclamation treatments increased the thermotolerance but decreased the fecundity of adults [15]. During the stress process, the expression of *Hsps* (*Hsp90*, *Hsc90*, *Hsp70*, and *Hsc70*) and ecdysone receptor genes (*EcRs*, *EcRA*, and *EcRB1*) were significantly upregulated. RNAi of *Hsc70* decreased the expression levels of two 20E-induced genes, *E74B* and *E75* [15]. In the present study, we found the expression of an ecdysone 20-monooxygenase-like gene (LOC118278003) was significantly downregulated in T1F1 males. A previous study on *S. frugiperda* also found that heat stress induced the upregulation of many Hsp-related genes and the ecdysone-induced protein 74EF-like gene (LOC118263008) in both male and female adults, as well as differential expression of many reproductive-related genes (19 up, 8 down) [31]. All the evidence together suggests that Hsps may be involved in the regulation of some signaling pathways, such as the 20 hydroxyecdysterone (20E) pathway, and the interaction between Hsps and EcRs or other proteins may serve as a bridge to regulate the trade-off between survival and reproduction under stresses. However, sex-specific heat and cold stress responses, such as metabolic processes, were observed in *S. frugiperda* adults [31]. Therefore, further research is needed to determine the possible adaptation mechanisms of female *S. frugiperda*.

GO enrichment analysis in our study also showed that DEGs were enriched to many more metabolism-related terms in each of the four comparison groups, with relatively more terms enriched in upregulated DEGs but fewer terms enriched in downregulated DEGs. This result is consistent with the result found in *S. frugiperda* males adults caused by short-duration heat stress [31]. In insects, the metabolic rate usually increases with increasing temperature [79]. However, heat stress may cause trade-offs between fundamental metabolic processes and stress-resistant processes [24] and protein denaturation, causing a decrease in metabolic rate [80]. Reversible protein phosphorylation is one of the main mechanisms for regulating metabolic rate, which is performed by multiple kinase proteins [81]. These kinases can inhibit or activate specific metabolic sites by regulating transcription factors, thereby regulating metabolic activity. A total of 34 stress response GO terms were enriched, with most (29) of them enriched in upregulated DEGs, including terms such as heat shock protein binding, response to abiotic stimulus, response to temperature stimulus, and response to heat.

KEGG enrichment analysis indicated that relatively more human disease- (52), metabolism- (25), and immunity-related (21) pathways were enriched in the four comparison groups, with most of them enriched in upregulated DEGs. Similarly, in *C. medinalis*, DEGs from both the heat-adapted and control larvae after heat stress were enriched to similar pathways related to longevity regulating, immunity, and diseases [74]. Further comparison of our results revealed that the number of disease pathways increased with the selection temperature in the first generation, which may be an indicator of stress cost, while in the T2 group, the number of disease pathways decreased obviously with subsequent generations, indicating enhanced acclimation.

Future research on important genes and pathways related to adaptation in both sexes will help achieve a deeper understanding of cold acclimation in insects.

## Data Availability

The original contributions presented in this study are included in the article/Supplementary Material. Further inquiries can be directed to the corresponding author.

## Supplementary Materials

The following supporting information can be downloaded at: https://www.mdpi.com/article/10.3390/insects16080860/s1, Figure S1: The principal component analysis (PCA) for RNA-seq of biological replicates of *S. frugiperda*. Figure S2: Volcanic plots of DEGs. (a–d) DEGs of T1F1-HS vs. CKF1-HS, T2F1-HS vs. CKF1-HS, T1F4-HS vs. CKF4-HS, and T2F4-HS vs. CKF4-HS, respectively. Significantly downregulated DEGs are indicated by blue dots, whereas significantly upregulated DEGs are indicated by red dots, with gray dots indicating genes with no significant differential expression. Figure S3: Venn diagram of DEGs. (a) Common DEGs within T1 groups; (b) common DEGs within T2 groups; (c) common DEGs within F1 groups; (d) common DEGs within F4 groups. The overlapping circles represent common DEGs among combinations. Figure S4: Heatmap of other resistance-related genes (other than Hsps) from the four comparison groups. T1F1, T2F1, T1F4, and T2F4 refer to T1F1-HS vs. CKF1-HS, T2F1-HS vs. CKF1-HS, T1F4-HS vs. CKF4-HS, and T2F4-HS vs. CKF4-HS, respectively. Table S1: Primers for real-time quantitative PCR. Table S2: Summary of the quality of all sample sequencing data. Table S3: Pearson’s correlation coefficient. Table S4: Temperature stress-induced DEGs. Table S5: GO enrichment analysis results. Table S6: KEGG enrichment analysis results. Table S7: Specific DEGs that may directly relate to temperature stress response and tolerance.
