# Supplementary figures and images for: Multigenerational Heat Selection Enhancing Thermal Acclimation and Transcriptional Response of Hsps to Heat Stress in Spodoptera frugiperda Male Adults
Source: Insects. 2025 Aug 18;16(8):860. doi: 10.3390/insects16080860 (PMC12386844; doi:10.3390/insects16080860)

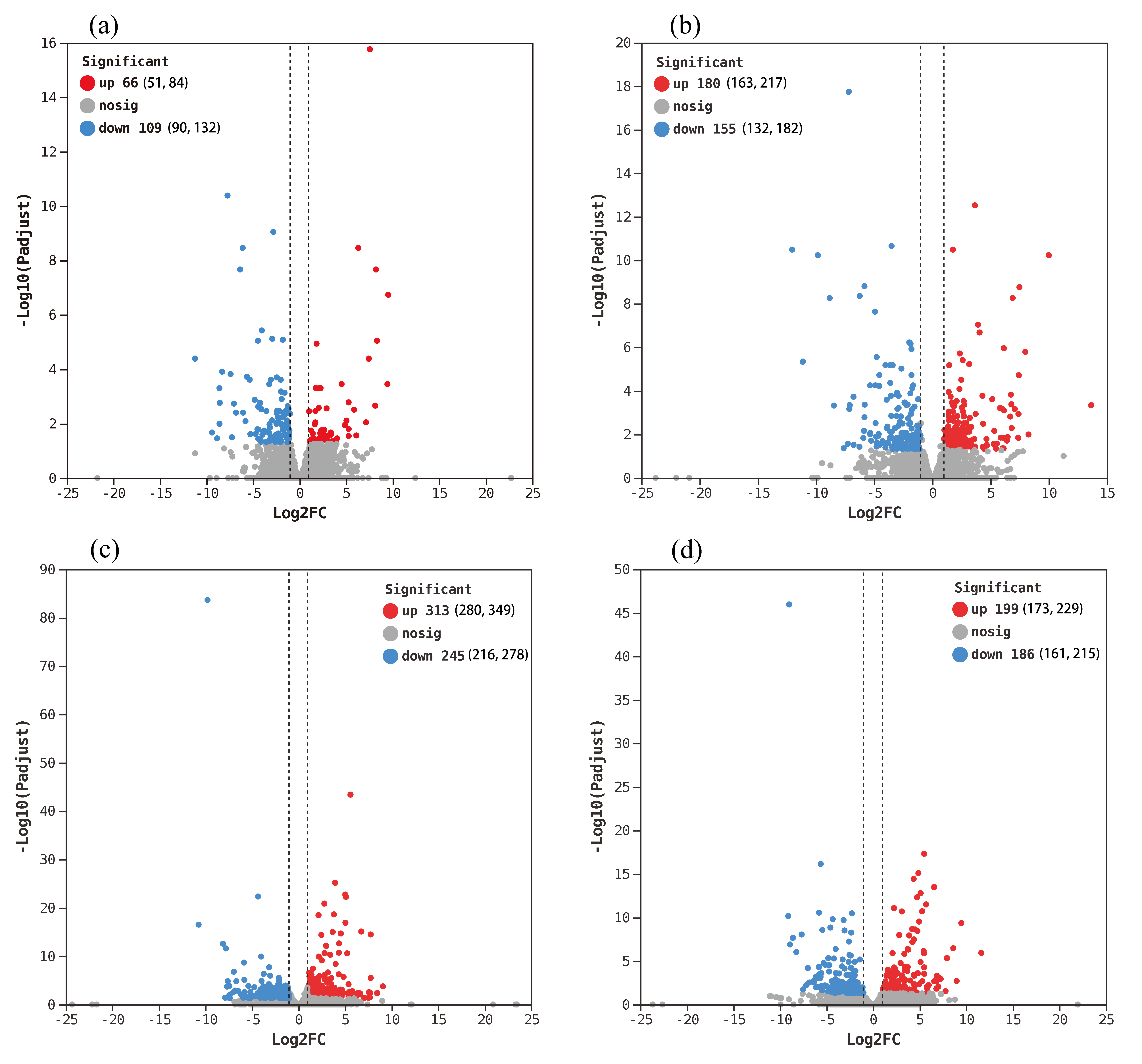

Supplement: Supplementary file 1 [file insects-16-00860-s001.zip › insects-3700500-supplementary/insects-3700500. Supplimental files/Figure S2.tif]

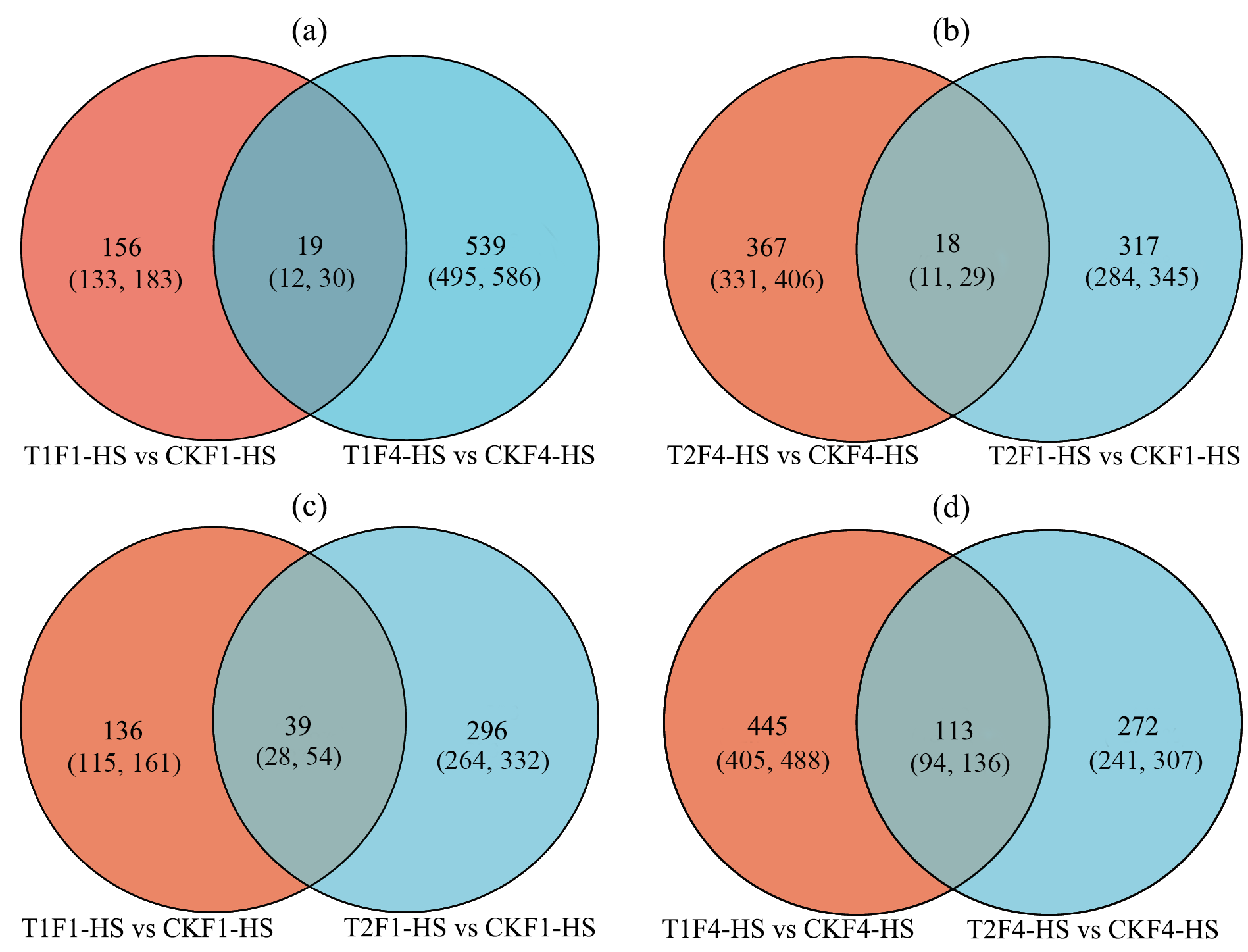

Supplement: Supplementary file 1 [file insects-16-00860-s001.zip › insects-3700500-supplementary/insects-3700500. Supplimental files/Figure S3.tif]

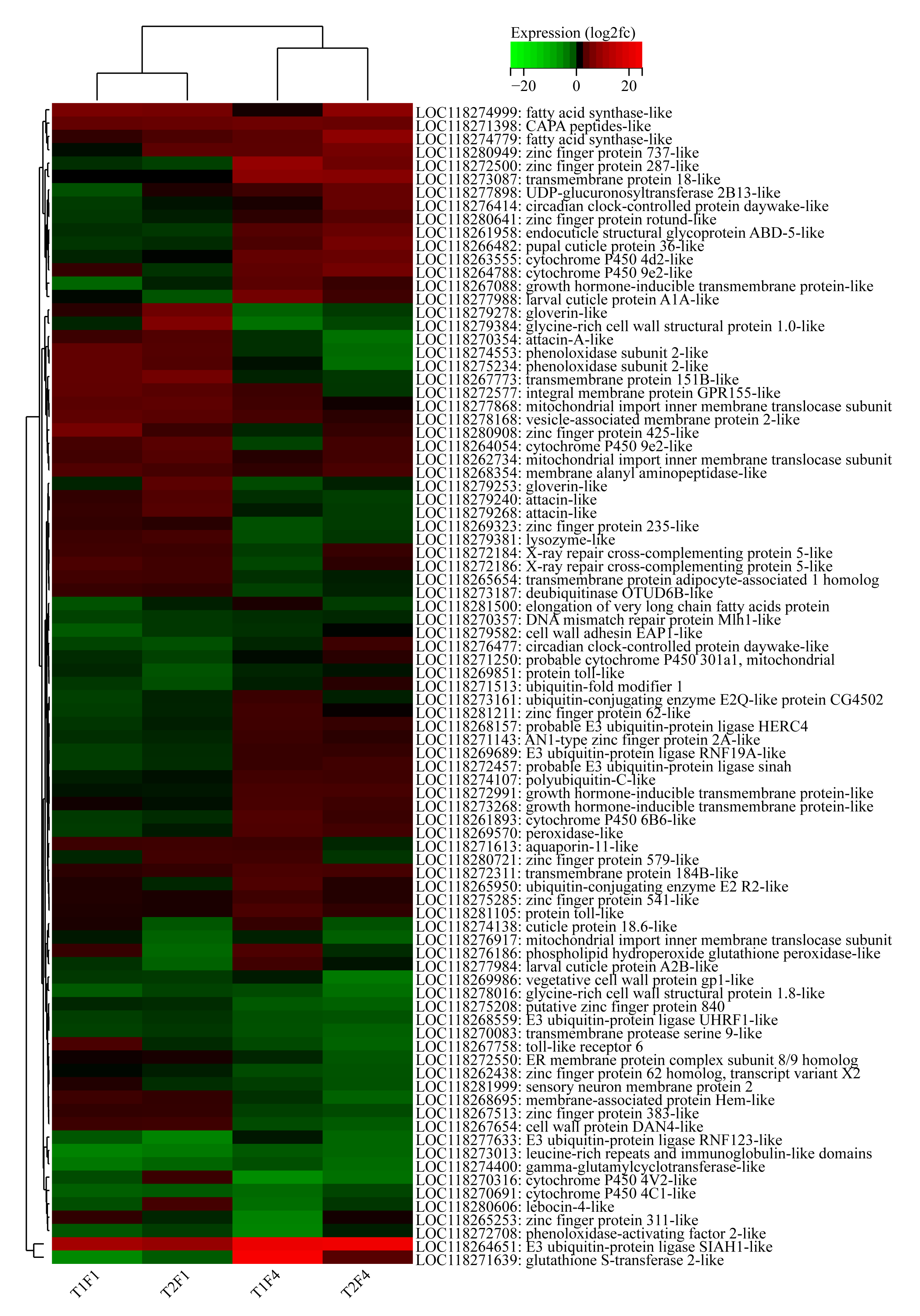

Supplement: Supplementary file 1 [file insects-16-00860-s001.zip › insects-3700500-supplementary/insects-3700500. Supplimental files/Figure S4.tif]
